# COVID-19–Associated Hospitalizations Among Health Care Personnel — COVID-NET, 13 States, March 1–May 31, 2020

**DOI:** 10.15585/mmwr.mm6943e3

**Published:** 2020-10-30

**Authors:** Anita K. Kambhampati, Alissa C. O’Halloran, Michael Whitaker, Shelley S. Magill, Nora Chea, Shua J. Chai, Pam Daily Kirley, Rachel K. Herlihy, Breanna Kawasaki, James Meek, Kimberly Yousey-Hindes, Evan J. Anderson, Kyle P. Openo, Maya L. Monroe, Patricia A. Ryan, Sue Kim, Libby Reeg, Kathryn Como-Sabetti, Richard Danila, Sarah Shrum Davis, Salina Torres, Grant Barney, Nancy L. Spina, Nancy M. Bennett, Christina B. Felsen, Laurie M. Billing, Jessica Shiltz, Melissa Sutton, Nicole West, William Schaffner, H. Keipp Talbot, Ryan Chatelain, Mary Hill, Lynnette Brammer, Alicia M. Fry, Aron J. Hall, Jonathan M. Wortham, Shikha Garg, Lindsay Kim, COVID-NET Surveillance Team, COVID-NET Surveillance Team, Nisha B. Alden, Kathy M. Angeles, Mirasol Apostol, David Blythe, Alicia Brooks, Susan Brooks, Sophrena Bushey, Erica Bye, Melissa Christian, Ashley Coates, Elizabeth Dufort, Nancy Eisenberg, Linda Frank, Maria Gaitan, Andrea George, Caroline Habrun, Emily B. Hancock, Brooke Heidenga, Kareena Hundal, Sarah A. Khanlian, RaeAnne Kurtz, Ruth Lynfield, Tiffanie Markus, Laine McCullough, Seth Meador, Alison Muse, Joelle Nadle, Meaghan Novi, Jake Ortega, Ama Owusu-Dommey, Rachel D. Park, Alexandra M. Piasecki, Andrea Price, Sherry Quach, Jeremy Roland, Maria Rosales, Yadira Salazar-Sanchez, Melanie Spencer, Ashley Swain, Michelle W. Wilson

**Affiliations:** ^1^CDC COVID-NET Team; ^2^Eagle Global Scientific, Atlanta, Georgia; ^3^Division of Healthcare Quality Promotion, National Center for Emerging and Zoonotic Infectious Diseases, CDC; ^4^California Emerging Infections Program, Oakland, California; ^5^Career Epidemiology Field Officer Program, CDC; ^6^Colorado Department of Public Health and Environment; ^7^Connecticut Emerging Infections Program, Yale School of Public Health, New Haven, Connecticut; ^8^Departments of Pediatrics and Medicine, Emory University School of Medicine, Atlanta, Georgia; ^9^Emerging Infections Program, Atlanta Veterans Affairs Medical Center, Atlanta, Georgia; ^10^Foundation for Atlanta Veterans Education and Research, Decatur, Georgia; ^11^Maryland Department of Health; ^12^Michigan Department of Health and Human Services; ^13^Minnesota Department of Health; ^14^New Mexico Emerging Infections Program, University of New Mexico, Albuquerque, New Mexico; ^15^New Mexico Department of Health; ^16^New York State Department of Health; ^17^University of Rochester School of Medicine and Dentistry, Rochester, New York; ^18^Ohio Department of Health; ^19^Public Health Division, Oregon Health Authority; ^20^Vanderbilt University Medical Center, Nashville, Tennessee; ^21^Salt Lake County Health Department, Salt Lake City, Utah.; Colorado Department of Public Health and Environment; New Mexico Emerging Infections Program; California Emerging Infections Program; Maryland Department of Health; Maryland Department of Health; California Emerging Infections Program; Rochester Emerging Infections Program; University of Rochester Medical Center; Minnesota Department of Health; New Mexico Emerging Infections Program; California Emerging Infections Program; New York State Department of Health; , New Mexico Emerging Infections Program; California Emerging Infections Program; Rochester Emerging Infections Program; University of Rochester Medical Center; Salt Lake County Health Department; New Mexico Emerging Infections Program; New Mexico Emerging Infections Program; California Emerging Infections Program; California Emerging Infections Program; New Mexico Emerging Infections Program; Rochester Emerging Infections Program; University of Rochester Medical Center; Minnesota Department of Health; Vanderbilt University Medical Center; Salt Lake County Health Department; CDC; New York State Department of Health; California Emerging Infections Program; New Mexico Emerging Infections Program; Salt Lake County Health Department; Public Health Division, Oregon Health Authority; Maryland Emerging Infections Program; The Johns Hopkins Bloomberg School of Public Health; CDC; Cherokee Nation Assurance; Salt Lake County Health Department; California Emerging Infections Program; California Emerging Infections Program; California Emerging Infections Program; New Mexico Emerging Infections Program; Salt Lake County Health Department; Salt Lake County Health Department; Maryland Emerging Infections Program; The Johns Hopkins Bloomberg School of Public Health.

Health care personnel (HCP) can be exposed to SARS-CoV-2, the virus that causes coronavirus disease 2019 (COVID-19), both within and outside the workplace, increasing their risk for infection. Among 6,760 adults hospitalized during March 1–May 31, 2020, for whom HCP status was determined by the COVID-19–Associated Hospitalization Surveillance Network (COVID-NET), 5.9% were HCP. Nursing-related occupations (36.3%) represented the largest proportion of HCP hospitalized with COVID-19. Median age of hospitalized HCP was 49 years, and 89.8% had at least one underlying medical condition, of which obesity was most commonly reported (72.5%). A substantial proportion of HCP with COVID-19 had indicators of severe disease: 27.5% were admitted to an intensive care unit (ICU), 15.8% required invasive mechanical ventilation, and 4.2% died during hospitalization. HCP can have severe COVID-19–associated illness, highlighting the need for continued infection prevention and control in health care settings as well as community mitigation efforts to reduce transmission.

COVID-NET conducts population-based surveillance for laboratory-confirmed COVID-19–associated hospitalizations among persons of all ages in 99 counties in 14 states (*1*). Hospitalized patients who are residents of the surveillance catchment area and have a positive SARS-CoV-2 molecular test result during their hospitalization or within 14 days before admission are included in COVID-NET. SARS-CoV-2 testing is performed at the discretion of health care providers or according to hospital testing policies. Trained surveillance officers conduct medical chart abstractions for COVID-19 patients using a standardized case report form, which includes HCP status. Data on HCP status collected by sites representing 98* counties in 13 states (California, Colorado, Connecticut, Georgia, Maryland, Michigan, Minnesota, New Mexico, New York, Ohio, Oregon, Tennessee, and Utah) are included in this analysis. HCP were defined as persons working in health care settings, home health care services, or health care occupations within other settings (e.g., school nurses) who have potential for exposure to patients or infectious materials (*2*). HCP were stratified into two groups for analyses according to presumed level of patient contact (i.e., those generally expected and those generally not expected to have direct patient contact) based on reported occupation.^†^

Because of high case counts, nine of 13 sites conducted in-depth medical chart abstractions for an age-stratified random sample of all reported COVID-19 patients hospitalized during March 1–May 31.^§^ Six sites completed chart abstractions for all patients aged <50 years (including all pregnant patients), 20% of patients aged 50–64 years, and 10% of patients aged ≥65 years. Three sites completed abstractions for 10% of patients aged ≥18 years, in addition to all pregnant patients. The remaining four sites completed chart abstractions for all reported patients. As of September 12, chart abstractions were complete for 86% of sampled patients identified through COVID-NET. Descriptive statistics were calculated for all sampled HCP aged ≥18 years hospitalized with COVID-19 during March 1–May 31, 2020, for whom full chart abstraction was completed. Weights were applied to reflect the probability of being sampled for complete chart abstraction; weighted percentages and unweighted case counts are presented throughout this report. Analyses were conducted using SAS (version 9.4; SAS Institute), and 95% confidence intervals (CIs) were generated using the Taylor series linearization method in SUDAAN (version 11; RTI International). COVID-NET activities were determined by CDC to meet the requirements of public health surveillance.^¶^ All sites participating in COVID-NET obtained approval from their respective state and local Institutional Review Boards, as applicable.

During March 1–May 31, 2020, COVID-NET received reports of 28,972 hospitalized adult patients, 8,515 of whom were sampled for complete chart abstraction (Figure 1). HCP status was documented for 6,760 sampled patients, 438 of whom were HCP, yielding a weighted estimate of 5.9% (95% CI = 5.1%–6.8%). The median age of HCP hospitalized with COVID-19 was 49 years (interquartile range [IQR] = 38–57 years), and 71.9% were female; 52.0% were non-Hispanic Black (Black), 27.4% were non-Hispanic White, and 8.6% were Hispanic or Latino persons (Table). More than two thirds (67.4%) of HCP hospitalized with COVID-19 worked in occupations in which they were generally expected to have direct patient contact; 36.3% of HCP hospitalized with COVID-19 worked in nursing-related occupations, including nurses (27.8%) and certified nursing assistants (CNAs) (8.5%). Patient aides and caregivers (6.6%) accounted for the next largest proportion of HCP hospitalized with COVID-19 (Figure 2).

**FIGURE 1:**
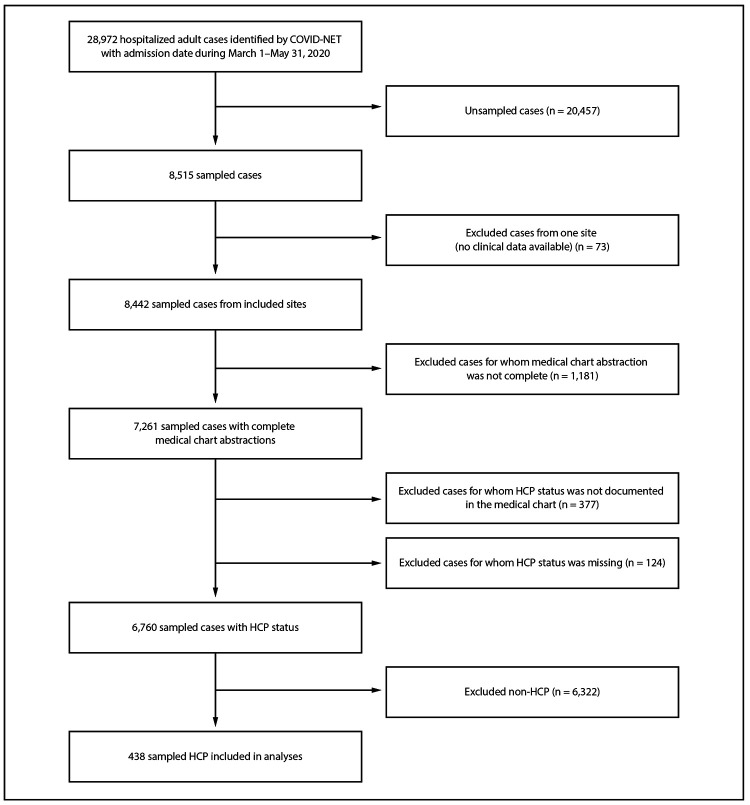
Selection of cases for analysis of COVID-19–associated hospitalizations among health care personnel (HCP)* — COVID-NET, 13 states,^†^ March 1–May 31, 2020 **Abbreviations:** COVID-19 = coronavirus disease 2019; COVID–NET = COVID–19–Associated Hospitalization Surveillance Network. * All case counts are unweighted. ^†^ Sites located in the following 13 states: California, Colorado, Connecticut, Georgia, Maryland, Michigan, Minnesota, New Mexico, New York, Ohio, Oregon, Tennessee, and Utah.

**TABLE:** Demographic and clinical characteristics of health care personnel (HCP) with COVID-19-associated hospitalizations, overall and by type of patient contact* — COVID-NET, 13 states,**^†^** March 1–May 31, 2020

Characteristic	Overall (N = 438)		Direct patient contact (N = 293)		No direct patient contact (N = 145)	
	Unweighted no. (weighted %)	95% CI	Unweighted no. (weighted %)	95% CI	Unweighted no. (weighted %)	95% CI
**Type of patient contact**						
Direct patient contact	293 (67.4)	(59.9–74.1)	—	—	—	—
No direct patient contact	145 (32.6)	(25.9–40.1)	—	—	—	—
**Age group (N = 438)**						
18**–**49 yrs	278 (46.4)	(39.1–53.7)	183 (44.4)	(35.7–53.4)	95 (50.5)	(37.6–63.4)
50**–**64 yrs	139 (46.1)	(38.9–53.5)	99 (51.0)	(42.0–59.9)	40 (36.0)	(24.7–49.2)
≥65 yrs	21 (7.5)	(4.1–13.3)	11 (4.7)	(2.0–10.7)	10 (13.4)	(5.9–27.7)
Median age in years (IQR)	49 (38–57)	—	52 (38–57)	—	48 (37–57)	—
**Race/Ethnicity (N = 438)**						
White, non-Hispanic	142 (27.4)	(21.5–34.1)	104 (33.3)	(25.5–42.2)	38 (15.0)	(8.9–24.3)
Black, non-Hispanic	184 (52.0)	(44.5–59.5)	113 (44.7)	(35.6–54.1)	71 (67.3)	(55.2–77.4)
Hispanic or Latino	48 (8.6)	(5.3–13.8)	30 (9.8)	(5.3–17.3)	18 (6.3)	(3.4–11.3)
American Indian or Alaska Native, non-Hispanic	39 (6.8)	(4.2–10.8)	29 (6.8)	(4.0–11.5)	10 (6.7)	(2.5–16.9)
Asian or Pacific Islander, non-Hispanic	12 (3.2)	(1.5–6.6)	10 (4.4)	(2.0–9.6)	2 (0.6)	(0.1–2.3)
Multiple races	1 (0.1)	(0.0–0.7)	1 (0.1)	(0.0–1.0)	—	—
Unknown	12 (1.9)	(0.7–4.9)	6 (0.8)	(0.4–1.9)	6 (4.1)	(1.1–14.1)
**Sex (N = 438)**						
Male	131 (28.1)	(21.8–35.3)	88 (29.5)	(21.8–38.6)	43 (25.2)	(15.6–37.9)
Female	307 (71.9)	(64.7–78.2)	205 (70.5)	(61.4–78.2)	102 (74.8)	(62.1–84.4)
**Underlying conditions (N = 438)**						
Any underlying condition^§^	377 (89.8)	(85.0–93.2)	248 (87.8)	(81.0–92.4)	129 (94.0)	(88.5–97.0)
Obesity (n = 396)	270 (72.5)	(65.2–78.7)	177 (68.3)	(58.8–76.4)	93 (80.9)	(70.3–88.4)
Hypertension	158 (40.6)	(33.5–48.2)	103 (36.9)	(28.6–46.1)	55 (48.3)	(35.4–61.4)
Chronic metabolic disease	136 (36.7)	(29.6–44.3)	88 (32.6)	(24.6–41.9)	48 (45.1)	(32.3–58.5)
Diabetes	115 (30.9)	(24.3–38.3)	72 (24.7)	(17.8–33.3)	43 (43.6)	(31.0–57.2)
Chronic lung disease	125 (26.7)	(20.6–33.9)	88 (26.6)	(19.5–35.3)	37 (26.9)	(16.6–40.6)
Asthma	92 (18.3)	(13.3–24.7)	66 (17.4)	(11.9–24.8)	26 (20.2)	(11.1–33.9)
Cardiovascular disease^¶^	45 (13.3)	(8.7–19.9)	27 (8.4)	(4.8–14.4)	18 (23.5)	(13.0–38.6)
Pregnancy (n = 189)**	34 (9.6)	(6.5–14.0)	22 (9.5)	(5.8–15.2)	12 (9.7)	(4.9–18.4)
Immunocompromised condition	28 (7.0)	(4.1–11.8)	17 (6.7)	(3.5–12.5)	11 (7.7)	(2.9–19.0)
**Signs and symptoms upon admission (N = 438)**						
Any symptoms	411 (96.6)	(94.4–98.0)	276 (96.4)	(93.1–98.1)	135 (97.1)	(94.5–98.5)
Shortness of breath	339 (79.0)	(72.0–84.5)	226 (77.9)	(69.1–84.7)	113 (81.2)	(68.8–89.5)
Cough	324 (76.6)	(69.7–82.3)	218 (75.1)	(66.2–82.3)	106 (79.8)	(68.6–87.7)
Fever/Chills	323 (73.9)	(66.7–80.1)	220 (75.0)	(66.0–82.2)	103 (71.8)	(58.6–82.2)
Muscle aches/Myalgias	177 (35.9)	(29.2–43.3)	126 (38.4)	(30.0–47.5)	51 (30.9)	(20.4–43.8)
Nausea/Vomiting	145 (31.6)	(25.0–39.1)	99 (33.8)	(25.5–43.1)	46 (27.2)	(17.3–40.1)
Headache	123 (29.3)	(22.8–36.7)	79 (27.6)	(20.0–36.8)	44 (32.8)	(21.7–46.2)
Diarrhea	114 (24.8)	(19.1–31.4)	75 (27.7)	(20.4–36.5)	39 (18.6)	(11.9–28.0)
Chest pain	105 (23.9)	(18.0–31.0)	67 (25.6)	(18.2–34.8)	38 (20.5)	(12.3–32.2)
Congested/Runny nose	65 (14.6)	(10.2–20.5)	46 (14.5)	(9.4–21.8)	19 (14.8)	(7.6–26.8)
Sore throat	66 (14.2)	(9.7–20.3)	51 (17.1)	(11.1–25.4)	15 (8.1)	(3.6–17.2)
Abdominal pain	46 (12.4)	(8.1–18.6)	32 (13.3)	(7.9–21.3)	14 (10.8)	(4.9–21.9)
Anosmia/Decreased smell	40 (9.4)	(5.7–15.1)	26 (11.4)	(6.3–19.7)	14 (5.2)	(2.6–10.1)
Dysgeusia/Decreased taste	36 (6.8)	(4.0–11.6)	20 (5.7)	(2.6–12.1)	16 (9.2)	(4.4–18.3)
Wheezing	29 (5.7)	(3.2–10.1)	19 (4.6)	(2.1–9.6)	10 (8.2)	(3.3–18.9)
**Hospital length of stay (median days, IQR)**	4 (3–9)	—	4 (2–9)	—	5 (3–9)	—
**Chest radiograph findings (N = 327)**						
Infiltrate/Consolidation	288 (86.9)	(79.3–92.0)	201 (91.4)	(84.3–95.5)	87 (76.8)	(58.9–88.4)
Bronchopneumonia/Pneumonia	84 (32.0)	(24.1–41.0)	58 (35.1)	(25.3–46.3)	26 (24.9)	(13.9–40.5)
Pleural effusion	11 (6.3)	(3.0–13.1)	5 (2.6)	(0.8–7.6)	6 (14.8)	(5.8–33.0)
**Chest CT/MRI findings (N = 94)**						
Infiltrate/Consolidation	56 (61.2)	(45.4–75.0)	38 (53.5)	(34.9–71.1)	18 (77.0)	(47.4–92.6)
Ground glass opacities	57 (59.9)	(44.0–73.9)	40 (61.4)	(42.4–77.5)	17 (56.7)	(29.5–80.3)
Bronchopneumonia/Pneumonia	41 (46.5)	(31.5–62.2)	29 (41.0)	(24.1–60.2)	12 (57.9)	(31.7–80.4)
Pleural effusion	10 (9.3)	(3.4–23.2)	9 (10.7)	(3.3–29.8)	1 (6.4)	(0.9–34.7)
**COVID–19 investigational treatments (N = 438)^††^**						
Received treatment	212 (48.2)	(40.8–55.8)	140 (47.5)	(38.5–56.7)	72 (49.8)	(36.8–62.8)
Hydroxychloroquine^§§^	152 (35.5)	(28.8–42.8)	96 (35.4)	(27.3–44.4)	56 (35.6)	(24.4–48.6)
Azithromycin^¶¶^	104 (25.9)	(19.8–32.9)	71 (25.6)	(18.6–34.2)	33 (26.3)	(16.2–39.8)
Remdesivir**^§§^**	54 (10.6)	(7.1–15.6)	43 (11.0)	(7.0–17.0)	11 (9.8)	(4.2–21.2)
Vitamins/minerals (i.e., vitamin C, zinc)	14 (8.9)	(5.0–15.6)	12 (10.4)	(5.4–19.0)	2 (6.0)	(1.5–20.8)
IL–6 inhibitors (i.e., tocilizumab, sarilumab)**^ §§^**	46 (8.2)	(5.6–12.0)	24 (5.6)	(3.2–9.4)	22 (13.7)	(7.8–23.0)
Convalescent plasma	19 (5.1)	(2.5–10.0)	14 (5.4)	(2.4–11.4)	5 (4.5)	(1.0–17.7)
Protease inhibitors (i.e., atazanavir, lopinavir/ritonavir)***	8 (1.7)	(0.6–4.3)	4 (0.6)	(0.2–1.5)	4 (4.0)	(1.2–12.5)
Other^†††^	8 (1.7)	(0.6–4.2)	8 (2.5)	(0.9–6.2)	—	—
**ICU admission (N = 438)**	116 (27.5)	(21.3–34.7)	80 (29.6)	(21.9–38.6)	36 (23.2)	(14.2–35.6)
ICU length of stay (median days, IQR)	6 (3–20)	—	6 (4–19)	—	5 (3–21)	—
**Interventions/Treatments (N = 438)^§§§^**						
Invasive mechanical ventilation^¶¶¶^	65 (15.8)	(11.1–22.0)	44 (15.6)	(10.2–23.1)	21 (16.3)	(8.5–28.9)
BIPAP/CPAP^¶¶¶^	13 (2.4)	(1.2–5.0)	9 (3.1)	(1.3–7.0)	4 (1.2)	(0.4–3.1)
High flow nasal cannula^¶¶¶^	28 (5.2)	(2.8–9.6)	21 (6.8)	(3.4–13.2)	7 (2.0)	(0.9–4.3)
Systemic steroids	74 (17.7)	(12.6–24.1)	47 (16.9)	(11.1–24.9)	27 (19.2)	(10.9–31.7)
Vasopressor (n = 436)	60 (14.4)	(10.0–20.3)	41 (15.2)	(9.9–22.8)	19 (12.8)	(6.4–23.9)
Renal replacement therapy	13 (2.1)	(1.0–4.6)	9 (1.9)	(0.8–4.8)	4 (2.6)	(0.7–9.7)
**Clinical discharge diagnoses (N = 438)**						
Pneumonia (n = 437)	213 (56.7)	(49.3–63.8)	148 (56.8)	(47.7–65.4)	65 (56.5)	(43.5–68.7)
Acute respiratory failure	170 (42.9)	(35.6–50.6)	117 (45.9)	(36.8–55.2)	53 (36.8)	(25.1–50.2)
Sepsis (n = 437)	63 (13.2)	(9.0–18.8)	44 (14.9)	(9.6–22.4)	19 (9.6)	(4.4–19.6)
Acute renal failure (n = 437)	46 (9.7)	(6.4–14.3)	28 (7.7)	(4.6–12.5)	18 (13.7)	(7.1–24.8)
Acute respiratory distress syndrome (n = 437)	38 (9.0)	(5.5–14.4)	24 (7.8)	(4.3–13.7)	14 (11.5)	(4.9–24.3)
Deep vein thrombosis (n = 159)	6 (7.4)	(2.9–17.4)	4 (7.9)	(2.7–21.0)	2 (6.3)	(1.0–30.1)
Pulmonary embolism (n = 159)	6 (6.0)	(2.5–14.0)	5 (7.7)	(2.9–18.8)	1 (2.5)	(0.3–15.8)
**Died during hospitalization (N = 438)**	16 (4.2)	(2.2–7.7)	11 (4.1)	(1.9–8.6)	5 (4.3)	(1.4–12.5)

**Abbreviations:** BIPAP = bilevel positive airway pressure; CI = confidence interval; COVID–19 = coronavirus disease 2019; COVID–NET = COVID–19–Associated Hospitalization Surveillance Network; CPAP = continuous positive airway pressure; CT = computed tomography; ICU = intensive care unit; IQR = interquartile range; MRI = magnetic resonance imaging.

* Reported HCP were categorized as those generally expected and those generally not expected to have direct patient contact based on HCP type.

^†^ Sites located in the following 13 states: California, Colorado, Connecticut, Georgia, Maryland, Michigan, Minnesota, New Mexico, New York, Ohio, Oregon, Tennessee, and Utah.

^§^ Defined as any of the following: chronic lung disease, chronic metabolic disease, blood disorder/hemoglobinopathy, cardiovascular disease, neurologic disorder, immunocompromised condition, renal disease, gastrointestinal/liver disease, rheumatologic/autoimmune/inflammatory condition, obesity (body mass index ≥30 kg/m^2^), and pregnancy.

^¶^ Excluding hypertension.

** Pregnancy was assessed among female patients aged 18–49 years; two pregnant patients were admitted to the ICU, and one required invasive mechanical ventilation.

^††^ Assessed as nonmutually exclusive treatment categories.

^§§^ Includes treatments administered as off–label, for compassionate use, or as part of randomized controlled trials (RCTs) for which the patient might have received treatment or a placebo: hydroxychloroquine (two), remdesivir (six), tocilizumab (one), and sarilumab (two).

^¶¶^ Given with at least one other COVID-19 investigational treatment.

*** Not given for human immunodeficiency virus infection.

^†††^ Eight patients received at least one of the following treatments: RCT for baricitinib (three), dexamethasone (three), cyclosporine (one), RCT for losartan (one), and RCT for LY3127804 (one).

^§§§^ Five (1.9%) patients received extracorporeal membrane oxygenation, and two (0.2%) received intravenous immunoglobulin.

^¶¶¶^ Highest level of respiratory support for each patient that needed respiratory support.

**FIGURE 2:**
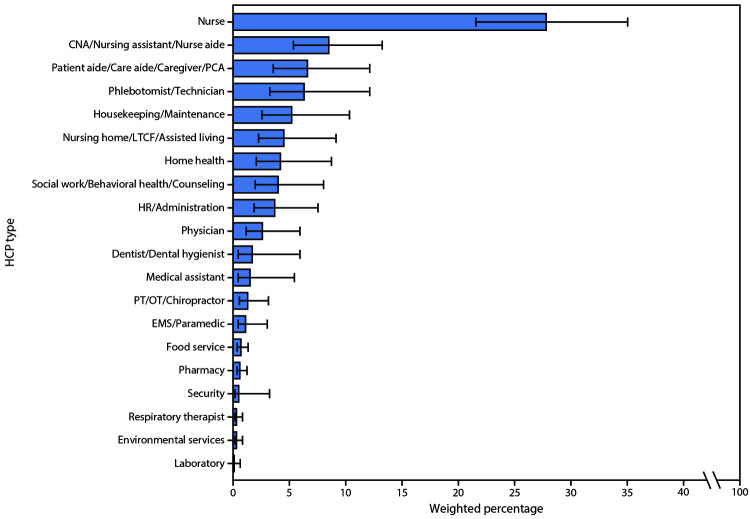
Weighted percentage of personnel types*,^†^ among reported health care personnel (HCP) with COVID-19–associated hospitalizations (N = 438) — COVID–NET, 13 states,^§^ March 1–May 31, 2020 **Abbreviations:** CNA = certified nursing assistant; COVID-19 = coronavirus disease 2019; COVID–NET = COVID–19–Associated Hospitalization Surveillance Network; EMS = emergency medical services; HR = human resources; LTCF = long-term care facility; OT = occupational therapist; PCA = patient care assistant; PT = physical therapist. * HCP categorized as “unspecified” or “other” have not been included in the figure but are included in the denominator. ^†^ Error bars represent 95% confidence intervals. ^§^ Sites located in the following 13 states: California, Colorado, Connecticut, Georgia, Maryland, Michigan, Minnesota, New Mexico, New York, Ohio, Oregon, Tennessee, and Utah.

Overall, 89.8% of HCP hospitalized with COVID-19 had documentation of at least one underlying condition (Table). The most commonly reported conditions included obesity (body mass index ≥30 kg per m^2^) (72.5%), hypertension (40.6%), and diabetes (30.9%). Compared with HCP generally expected to have direct patient contact, those generally not expected to have direct patient contact had higher prevalences of obesity (80.9% versus 68.3%) and cardiovascular disease (excluding hypertension) (23.5% versus 8.4%). Among female HCP aged 18–49 years hospitalized with COVID-19, 9.6% were pregnant during hospitalization. Upon hospital admission, 96.6% of HCP reported COVID-19–associated signs and symptoms; shortness of breath (79.0%), cough (76.6%), and fever or chills (73.9%) were those most commonly reported.

The median length of hospitalization among HCP with COVID-19 was 4 days (IQR = 3–9 days). COVID-19 investigational treatments were administered to 48.2% of HCP hospitalized with COVID-19. Overall, 27.5% of HCP were admitted to an ICU for a median of 6 days (IQR = 3–20 days), and 15.8% required invasive mechanical ventilation. Pneumonia was a documented discharge diagnosis for 56.7% of HCP hospitalized with COVID-19 and acute respiratory failure for 42.9%. Sixteen (4.2%) HCP with COVID-19 died during hospitalization.

## Discussion

During March 1–May 31, 2020, HCP accounted for approximately 6% of adults hospitalized with COVID-19 for whom HCP status was documented in COVID-NET. The median age of hospitalized HCP (49 years) was substantially lower than that previously reported for hospitalized adults (62 years) (*3*). More than two thirds (67.4%) of HCP hospitalized with COVID-19 were generally expected to have direct patient contact, and over one third (36.3%) were in nursing-related occupations. Similar to the proportion of underlying conditions among all hospitalized adults reported to COVID-NET during March–May,** approximately 90% of hospitalized HCP reported at least one underlying condition, with obesity being the most common and reported for over two thirds (72.5%) of patients. A high proportion of hospitalized HCP had indications of severe disease: approximately one in four were admitted to an ICU, and approximately 4% died. The proportion of HCP with these severe clinical outcomes was similar to that of adults aged 18–64 years hospitalized with COVID-19 during March–May.^††^

Findings from this analysis are comparable to those reported among HCP with COVID-19 in China, which found that nursing-related occupations accounted for the largest proportion of COVID-19 cases among HCP (*4*). COVID-NET does not specifically collect information on exposure history; however, nurses are frontline workers and might be at particular risk for exposure because of their frequent and close patient contact, leading to extended cumulative exposure time. Nursing-related occupations also account for a large proportion of the U.S. health care workforce: in 2019, registered nurses alone represented approximately one third of health care practitioners (*5*). This has implications for the capacity of the health care system, specifically nursing staff members, to respond to increases in COVID-19 cases in the community. To decrease the risk for SARS-CoV-2 transmission in health care facilities, CDC recommends that HCP use face masks (i.e., medical masks, such as surgical or procedure masks) at all times while they are in health care facilities, including patient-care areas, staff member rooms, and areas where other HCP might be present (*2*). In addition, in areas with moderate to substantial community transmission of SARS-CoV-2, CDC recommends that HCP wear eye protection for all patient care encounters. An N95-equivalent or higher-level respirator is recommended for aerosol-generating procedures and certain surgical procedures to provide optimal protection against potentially infectious respiratory secretions and aerosols (*2*).

Similar to the distribution of the U.S. health care workforce overall, a majority of hospitalized HCP in this report were female (*5*). However, compared with previously reported demographic characteristics of U.S. HCP with COVID-19, HCP identified by COVID-NET were older, and a larger proportion were Black (*6*). Given that COVID-NET conducts surveillance specifically for hospitalized patients, these differences might reflect the association between increased age and severe outcomes associated with SARS-CoV-2 infection as well as disproportionate effects among Black populations (*1*,*3*,*7*,*8*).

These results are consistent with previously reported data suggesting that underlying conditions, including obesity, diabetes, and cardiovascular disease, are risk factors for COVID-19–associated hospitalization and ICU admission (*3*,*9*,*10*). Among the approximately 90% of HCP in this analysis with at least one underlying condition, obesity was most commonly reported. A recent study found that obesity was highly associated with risk for death among COVID-19 patients who sought health care, even after adjusting for other obesity-related underlying conditions (*10*). The findings in this report highlight the need for prevention and management of obesity through evidence-based clinical care as well as policies, systems, and environmental changes to support HCP in healthy lifestyles to reduce their risk for poor COVID-19–related outcomes.^§§^

The findings in this report are subject to at least five limitations. First, HCP status is determined through medical chart review, and although chart abstractions will be completed on all sampled cases, abstraction was pending at the time of analysis for approximately 14% of sampled cases hospitalized during March–May. Thus, the proportion of identified HCP among all adults hospitalized with COVID-19 from March–May might represent an overestimate or underestimate of HCP in this population. Second, because of small sample sizes for some variables, some estimates might be unstable, as evidenced by wider confidence intervals. Third, although COVID-NET collects HCP status, data on the degree, frequency, and duration of contact with patients are not collected. HCP were stratified by presumed level of patient contact, based on general understanding of health care professions; the level of patient contact for some HCP might have thus been misclassified. Fourth, COVID-NET does not collect data regarding exposure history. It is unknown whether HCP were exposed to SARS-CoV-2 in the workplace or community, highlighting the need for community prevention efforts as well as infection prevention and control measures in health care settings. Finally, laboratory confirmation is dependent on clinician-ordered testing and hospital testing policies for SARS-CoV-2; as a result, COVID-19–associated hospitalizations might have been underestimated.

Findings from this analysis of data from a multisite surveillance network highlight the prevalence of severe COVID-19–associated illness among HCP and potential for transmission of SARS-CoV-2 among HCP, which could decrease the workforce capacity of the health care system. HCP, regardless of any patient contact, should adhere strictly to recommended infection prevention and control guidance at all times in health care facilities to reduce transmission of SARS-CoV-2, including proper use of recommended personal protective equipment, hand hygiene, and physical distancing (*2*). Community mitigation and prevention efforts in households and congregate settings are also necessary to reduce overall SARS-CoV-2 transmission. Continued surveillance of hospitalized HCP is necessary to document the prevalence and characteristics of COVID-19 among this population. Further understanding of exposure risks for SARS-CoV-2 infection among HCP is important to inform additional prevention strategies for these essential workers.

**Summary**

**What is already known about this topic?**

Data on characteristics and outcomes of U.S. health care personnel (HCP) hospitalized with COVID-19 are limited.

**What is added by this report?**

Analysis of COVID-19 hospitalization data from 13 sites indicated that 6% of adults hospitalized with COVID-19 were HCP. Among HCP hospitalized with COVID-19, 36% were in nursing-related occupations, and 73% had obesity. Approximately 28% of these patients were admitted to an intensive care unit, 16% required invasive mechanical ventilation, and 4% died.

**What are the implications for public health practice?**

HCP can have severe COVID-19–associated illness, highlighting the need for continued infection prevention and control in health care settings as well as community mitigation efforts to reduce SARS-CoV-2 transmission.

## Footnotes

* Counties represented in analysis: California (Alameda, Contra Costa, and San Francisco counties); Colorado (Adams, Arapahoe, Denver, Douglas, and Jefferson counties); Connecticut (Middlesex and New Haven counties); Georgia (Clayton, Cobb, DeKalb, Douglas, Fulton, Gwinnett, Newton, and Rockdale counties); Maryland (Allegany, Anne Arundel, Baltimore, Baltimore City, Calvert, Caroline, Carroll, Cecil, Charles, Dorchester, Frederick, Garrett, Harford, Howard, Kent, Montgomery, Prince George’s, Queen Anne’s, St. Mary’s, Somerset, Talbot, Washington, Wicomico, and Worcester counties); Michigan (Clinton, Eaton, Genesee, Ingham, and Washtenaw counties); Minnesota (Anoka, Carver, Dakota, Hennepin, Ramsey, Scott, and Washington counties); New Mexico (Bernalillo, Chaves, Doña Ana, Grant, Luna, San Juan, and Santa Fe counties); New York (Albany, Columbia, Genesee, Greene, Livingston, Monroe, Montgomery, Ontario, Orleans, Rensselaer, Saratoga, Schenectady, Schoharie, Wayne, and Yates counties); Ohio (Delaware, Fairfield, Franklin, Hocking, Licking, Madison, Morrow, Perry, Pickaway, and Union counties); Oregon (Clackamas, Multnomah, and Washington counties); Tennessee (Cheatham, Davidson, Dickson, Robertson, Rutherford, Sumner, Williamson, and Wilson counties); and Utah (Salt Lake County).

† HCP generally expected to have direct patient contact included nurse (115), CNA/nursing assistant/nurse aide (50), patient aide/care aide/caregiver/patient care assistant (25), home health personnel (17), phlebotomist/technician (16), social work/behavioral health/counseling (16), physician (15), physical therapist/occupational therapist/chiropractor (nine), dentist/dental hygienist (seven), emergency medical services personnel/paramedic (seven), medical assistant (six), nursing home/long-term care/assisted living staff members (three), respiratory therapist (three), and other (four). HCP generally not expected to have direct patient contact included human resources/administrative staff members (22), housekeeping/maintenance staff members (13), nursing home/long-term care/assisted living staff members, role unspecified (12), food service (seven), pharmacist/pharmacy staff members, role unspecified (six), environmental services (three), laboratory staff members, role unspecified (one), security (one), other (five), and unspecified (75). HCP categorized as “role unspecified” were those for whom only a location of work was indicated with no other detail about occupation; all such HCP were assumed generally not to have direct patient contact and were classified according to their location of work.

§ https://www.cdc.gov/coronavirus/2019-ncov/covid-data/covidview/purpose-methods.html.

¶ 45 C.F.R. part 46, 21 C.F.R. part 56; 42 U.S.C. **241(d); 5 U.S.C. **552a; 44 U.S.C. **3501 et seq.** https://gis.cdc.gov/grasp/COVIDNet/COVID19_5.html.

†† https://gis.cdc.gov/grasp/COVIDNet/COVID19_5.html.

§§ https://www.cdc.gov/obesity/strategies/index.html.
