# *Erratum*: Vol. 69, No. 43

**DOI:** 10.15585/mmwr.mm6945a8

**Published:** 2020-11-13

In the report “COVID-19–Associated Hospitalizations Among Health Care Personnel — COVID-NET, 13 States, March 1–May 31, 2020,” on page 1579, in the Table, the row headings for rows 13 and 14 were incorrect and should have appeared as below:

**Table:** 

**Asian or Pacific Islander, non-Hispanic**	39 (6.8)	(4.2–10.8)	29 (6.8)	(4.0–11.5)	10 (6.7)	(2.5–16.9)
**American Indian or Alaska Native, non-Hispanic**	12 (3.2)	(1.5–6.6)	10 (4.4)	(2.0–9.6)	2 (0.6)	(0.1–2.3)

